# Regulation of Seed Dormancy and Germination Mechanisms in a Changing Environment

**DOI:** 10.3390/ijms22031357

**Published:** 2021-01-29

**Authors:** Ewelina A. Klupczyńska, Tomasz A. Pawłowski

**Affiliations:** Institute of Dendrology, Polish Academy of Sciences, Parkowa 5, 62-035 Kórnik, Poland; evelin@man.poznan.pl

**Keywords:** adaptation, climate change, gene expression, plasticity, reproduction

## Abstract

Environmental conditions are the basis of plant reproduction and are the critical factors controlling seed dormancy and germination. Global climate change is currently affecting environmental conditions and changing the reproduction of plants from seeds. Disturbances in germination will cause disturbances in the diversity of plant communities. Models developed for climate change scenarios show that some species will face a significant decrease in suitable habitat area. Dormancy is an adaptive mechanism that affects the probability of survival of a species. The ability of seeds of many plant species to survive until dormancy recedes and meet the requirements for germination is an adaptive strategy that can act as a buffer against the negative effects of environmental heterogeneity. The influence of temperature and humidity on seed dormancy status underlines the need to understand how changing environmental conditions will affect seed germination patterns. Knowledge of these processes is important for understanding plant evolution and adaptation to changes in the habitat. The network of genes controlling seed dormancy under the influence of environmental conditions is not fully characterized. Integrating research techniques from different disciplines of biology could aid understanding of the mechanisms of the processes controlling seed germination. Transcriptomics, proteomics, epigenetics, and other fields provide researchers with new opportunities to understand the many processes of plant life. This paper focuses on presenting the adaptation mechanism of seed dormancy and germination to the various environments, with emphasis on their prospective roles in adaptation to the changing climate.

## 1. Introduction

Seeds evolve from the very beginning of their existence to become efficient environmental change detectors, responding to their prevailing conditions, and also being characterized by specific variations depending on the past fluctuations in the environment that also affect the construction and variability of future seeds [1,2,3]. One of the adaptations to unfavorable environmental conditions is the dormancy phenomenon, which allows the coordination of seed germination and plant establishment with the environment. In ecological and evolutionary terms, this property is of paramount importance and is indispensable for preserving species continuity and maintaining biodiversity [4]. As one of the earliest features expressed in the plant life cycle, seed dormancy can be a critical determinant of colonization and distribution of a species [4,5]. The regulation of dormancy and initiation of germination allow plants to survive in unfavorable conditions of the environment in which they live [6,7].

Global climate change is currently affecting environmental conditions and changing the reproduction of plants from seeds. The appearance of disturbances in the phenology of germination will cause disturbances in the diversity of plant communities [8]. Climate change may alter the geographical distribution of species, with the plasticity of the species being the determining factor for the survival of populations.

In response to the climate, seed dormancy has changed, both in the long term as well as in the short term. Long-term effects emerge from sustained climatic differences among sites, which may result in inheritable dormancy differences through species, ecotype, and clinal variation [9]. On the other hand, short-term effects are produced by the specific weather during the seed maturation season [10,11,12], during seed storage in the soil, and during seed germination. Understanding and explaining how seed dormancy reacts to the local climate in the short- and long-term is critical to understanding how changes in germination and plant regeneration are affected by environmental changes, including climate warming [8,13,14].

The environmental conditions to which the plants are exposed during seed maturation, usually termed the parental or maternal environment effect, affect the dormancy level and germination time [15,16,17,18]. Lower temperatures acting on the maternal plant tend to increase the depth of seed dormancy [10,19,20,21]. However, the quantitative relationship between temperature during seed development and the level of dormancy showed that a positive linear relationship exists between the average temperature and the germination index [22]. Other environmental factors experienced during seed maturation—e.g., water stress [23] or the availability of nutrients, in particular nitrate—also affect depth of dormancy [20,24,25]. Understanding these genetics and environment interactions for different seeds is important to aid breeding for adequate dormancy responses under particular environments [22].

After harvest, dormant seeds may remain so for a long period [19,26]. During this time, they constantly adjust their dormancy states by detecting and integrating many environmental signals [27]. Soil temperature and humidity are the environmental factors that control the depth of dormancy in mature seeds, being the basic factors responsible for dormancy cycling [1,28,29]. The rate of increase and decrease in seed dormancy during the year is controlled by seasonal changes in soil parameters. In temperate soils, temperature and humidity signals occur as slow seasonal changes that indicate the appropriate time of year for germination and seedling establishment (i.e., a temporal window) [30,31,32,33,34]. To change the depth of dormancy, these signals are integrated in time and, consequently, also change the sensitivity of seeds to the second set of signals that remove dormancy and allow germination to end (e.g., light, smoke, nitrate, alternating temperatures) [34,35,36,37]. The second set of signals indicates in a more direct manner that the conditions are suitable for ending the dormancy period and for terminating germination (i.e., a spatial window). If the correct spatial window does not appear (i.e., favorable habitat conditions), the time window will close for the following year [7]. Dormancy aligns with the seasons, thus determining the optimal time of plant establishment and enabling the spreading of a population’s germination events through time [38].

Many physiological and molecular mechanisms that regulate dormancy have been identified individually in controlled laboratory studies [27]. However, little is known about how seeds use this complex suite of mechanisms to regulate dormancy in a variable environment. Understanding the response of plants in response to climate change requires more extensive knowledge on the impact of environmental conditions on seed dormancy and germination, including ecological and physiological/molecular sites. The mechanism underlying climate adaptation is a key element in predicting the potential of species to face climate warming [39,40,41]. Integrating research techniques from different disciplines of biology could aid understanding of the mechanisms of the processes controlling seed germination [42,43].

## 2. Adaptations of Seed Germination to the Changing Environment

**Figure 1:**
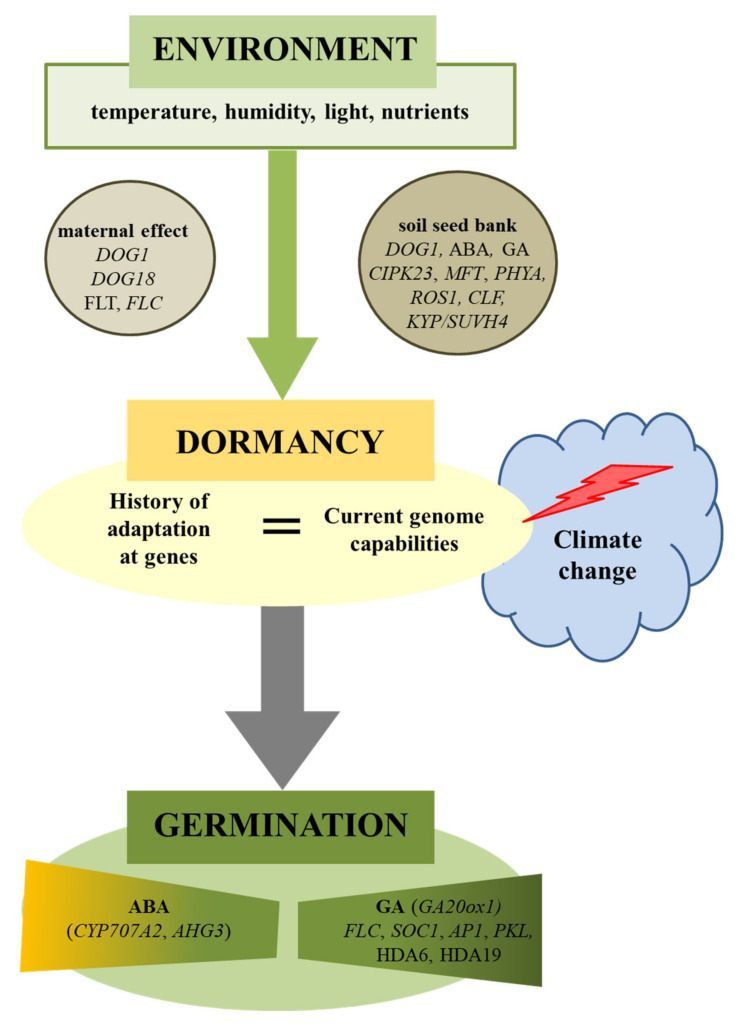
Environmental regulation of plant seed dormancy and germination. Seed germination is dependent on environmental conditions acting during maturation (maternal effect) as well as during storage (soil seed bank). Climate change can cause disturbances in the seed germination if the history of species (genetics) as well as the present (ecology) will not match. Physiological and molecular factors were involved in this regulation. ABA-hypersensitive Germination 3 (AHG3), abscisic Acid (*ABA*), Apetala1 (*AP1*), Cbl-Interacting Protein Kinase 23 (*CIPK23*), Curly Leaf (*CLF*), Cytochrome P450 Monooxygenase (*CYP707A2*), Delay OF Germination 1 (*DOG1*), Flowering Locus T (*FLT*), Flowering Locus C (*FLC*), Giberellic Acid (*GA*), Gibberellin 20-Oxidase 1 (*GA20ox1*), Histone Deacetylase 6 (*HDA6*), Kryptonite (*KYP/SUVH4*), Mother OF Flowering Time (*MFT*), Nitrite Reductase 1 (*NRT1.1*), Pickle (*PKL*), Phytochrome A (*PHYA*), Repressor OF Silencing1 (*ROS1*), Suppressor of Overexpression of Constans 1 (*SOC1*).

Climate and geographical changes in the history of a population are important indicators of the adaptive evolution of seed germination [4,44]. A meta-analysis of 3164 plant species showed that plants in environments associated with frost and/or drought are more likely to have some form of dormancy [45]. The environmental variability (seasonality) is crucial to explaining the existence or absence of dormancy and evolutionary transitions between these states [46]. In addition to the genetic background of this adaptation, environmental factors can shape the depth of dormancy and seed germination [8,47]. Models developed for climate change scenarios show that many species would face a significant decrease in suitable habitat area [48], also because of disturbances in the reproduction process (Figure 1). For example, in species with physiological dormancies, germination will be delayed with global warming, if the current length of the stratification period approximates its minimum requirement, as shortened winters will not adequately overcome dormancy. On the other hand, germination will be earlier if the current length of stratification greatly exceeds the minimum required, as premature spring warm-up accelerates germination [8]. Simulation of the germination response to diurnally alternating temperatures under climate change scenarios showed that increasing temperatures decreased the base temperature for seed germination and the thermal time required for germination [49]. The effect of higher temperatures increased germination under future climate scenarios, but this seems to be only to a limited extent—temperature will also have inhibitory effects [50,51]. The germination variability, however, complicates generalizing the impact of climate change.

The number of studies conducted on the ecophysiology of seed dormancy and germination with regard to climate change is now increasing [52]. Changes in temperature and water availability affect the seed germination and survival of plants [53,54]. Germination responses to temperature differ among species but also within species across their latitudinal and altitudinal ranges [55]. In response to environmental factors, seed dormancy and germination characteristics may vary within one species. Many authors have demonstrated variability of dormancy among seed collections from different places and years [56,57,58,59]. Variability of dormancy shows latitudinal differences on a wide geographical scale [60,61,62], and there is also a positive correlation between dormancy and population altitude [14,63,64,65]. Variability of dormancy has also been detected among seed collections from different environments—e.g., the negative influence of winter temperatures on seed dormancy in a wide gradient along western North America has been described for *Artemisia tridentata* [66]. Differences in the germination ability of *Thymelaea hirsuta* (L.) Endl. seeds collected from six different desert habitats has been established, with lower germination in seeds from more extreme sites [67]. Seed dormancy responses to temperature in Patagonian *Nothofagus* species are related to distribution and determines temporal patterns of germination across altitudes [68]. This phenomenon may influence the maintenance of vegetation patterns in these ecosystems by placing germinated seeds in a favorable environment for growth. It is presumed that the mere presence of intraspecies variability shows the enormous potential of physiological dormancy in adaptation to rapid environmental changes [14].

Understanding the sensitivity of a given species to changes in climate requires determining its germination temperature thresholds, including the variations among populations along the climate gradient they populate [55]. The responses are usually idiosyncratic and related to the local climate of the population. For instance, in studies along latitudinal gradients, in some cases, an inverse relationship between latitude and germination temperature has been documented, whereby populations at higher latitudes have increased germination rates at warmer temperatures, while populations at lower latitudes have high germination rates at colder temperatures. However, other works show the opposite pattern or the lack of a clear relationship between latitude and germination temperature [55]. Germination of western Iberia *Erica australis* and *E. umbellata* seeds showed that rising temperatures will affect these species, particularly at their northern ranges, where many seeds will remain dormant during warmer winters [69]. This study proved that models aiming at assessing climate change impacts in the species need to include the variability across latitudes. The examination of the species from arid Australia showed that some of the studied species had significantly greater levels of germination after exposure to predicted increased soil temperatures, in addition to another species displaying a dramatic decrease in seed viability [52]. Additionally, Dwyer and Erickson [70] indicate that most of the Australian (the Mediterranean climate) winter annual species studied will germinate higher fractions of seeds under future climate conditions due to the cumulative effects of warmer maternal, after-ripening, and germination environments. The analysis of 55 cactus species from the Americas, reflecting the broad environmental envelope of the family, indicated that 25% of species will have reduced germination performances, whilst the remainder will have efficiency gains by the end of the 21st century [71]. In the example of species from a Brazilian tropical dry forest which is tolerant to extreme temperature and water deficits, in future climate scenarios, rainfall rather than temperature will be extremely limiting for seed germination [72]. The same was showed for savanna species *Acacia nigrescens* and *Colophospermum mopane*, where higher future temperatures will not limit germination directly, but they will reduce the number of germination events by reducing the time window of suitable available soil water [73].

Studies on Atlantic–European *Hypericum elodes* L., characterized by physiological dormancy, showed that the populations did not respond equally to stratification, as the benefit of cold stratification for the southern population was lower [74]. The seed dormancy was clearly correlated with the maturation environment: higher temperatures in the summer and winter precipitation predicted poor and rapid loss of dormancy, respectively. Research on the impact of autumn and spring heat waves on the seed germination of alpine plants showed that, in the absence of heat waves, germination took place mainly in spring, but in autumn, it was the opposite—germination increased significantly after heat waves in half of the tested species [75]. The study showed that heat waves can affect germination time, and warming can lead to germination transition mostly from spring to autumn [76], especially among nondormant or conditionally dormant seeds [26]. *Vitis vinifera* subsp. *sylvestris* was investigated in four Sardinian populations over the full altitudinal range of the species [77]. Under the simulated climate warming scenarios, an altitude-related risk from climate warming was identified, with lowland populations being more threatened due to a compromised seed dormancy release and a narrowed seed germination window. Daws et al. [18] observed differences in the dormancy breakage and germination of seeds collected from sycamore trees growing in Europe. Batches of seeds from the south germinated under the influence of higher temperatures than those from the north, and batches of seeds from northern Europe required low temperature stratification. The effect on the germination had the sum of heat to which the trees were exposed during seed maturation.

Turkish pine (*Pinus brutia* Ten.) seeds growing in a cold climate germinated only under the influence of low temperatures, whereas seeds of trees growing in a dry, warm climate germinated at a wide temperature range [61]. Their adaptation strategy (survival of young seedlings) assumes the germination of seeds in the spring (cold climate) or in autumn (hot and dry climate), or both seasons (in intermediate conditions). Gosling et al. [78], who studied black alder [*Alnus glutinosa* (L.) Gaertn.] seeds, showed that cold stratification improves seed germination in a wider temperature range. This feature promotes germination in the autumn and stimulates earlier and more synchronous seed emergence in a wider range of temperatures in the next spring. This also shows how the population can survive climate changes. If climate change brings a longer and warmer autumn, more seeds will germinate before winter. If the winter will be warm and/or short, those seedlings will develop well until the spring. Even if frost kills these seedlings, some seeds will remain dormant until the spring. These experiments showed the adaptive potential of black alder to the changing climate.

Research conducted on the Mediterranean genus *Romulea* shows that phylogenetically closely related species show differences in ecophysiological traits, such as dormancy and germination characteristics, thus reflecting the different habitats and bioclimatic areas in which they occur [79]. The authors stated that the seed maturation environment may play an even greater role than genetic variation in explaining the processes of dormancy [80]. Vidigal et al. [79] linked the seed dormancy of *A. thaliana* (from different stands in Spain) with altitude and climate. They indicated that deep dormancy is associated with high temperature, low rainfall, and high sunlight. Escudero et al. [81] studied different pine species and found intraspecific and intrapopulation variability in seed germination. The authors concluded that the differences between populations are not a consequence of different ecotypes, but are the result of the environment of the mother plant and the maternal genotype. Variability in seed germination may increase the chances of survival of the species under changing climatic conditions [82]. Another study on *A. thaliana* showed that both the origin of the population and the temperature during seed maturation affect the level of dormancy and the expression of the genes controlling dormancy variability [83]. A study of the dormancy of *Centaurium somedanum* Lainz. seeds showed that populations growing at lower altitudes in a generally milder climate benefited from a longer growing season and produced seeds that would sprout earlier [14]. Plants from higher heights, where the winters are sharper, produced seeds that would not germinate until the end of the unfavorable season. The authors also proved that dormancy has a genetic basis, but may show significant adaptive changes in response to short-term climate changes [14].

Climate change may be beneficial for some plant species, enabling them to find new ecological niches, which have had so far unfavorable conditions for the production and germination of seeds. Global warming may increase germination capacity and seed survival of species characterized by high plasticity. It seems, however, that along with the deepening of the climate changes, as a result of exceeding the tolerance barriers for a given species, the developed adaptive mechanisms related to reproduction of plants may fail, consequently leading to the disappearance of species in a given area. From a practical point of view, it seems necessary to conduct new experiments in natural conditions on the influence of climate on the germination of seeds, which will enable the determination of the relationship between the reproduction of plants from seeds and climate warming and facilitate the selection of appropriate, more plastic populations and species.

## 3. Mechanisms of Adaptation of Seed Germination to the Changing Environment

A variety of dormancy mechanisms have been observed, in line with the diversity of climates and habitats that various plant species have been able to colonize [84,85]. Physiological dormancy is the most commonly occurring form across all major angiosperm clades and is the class present in most seed model species [1,27,85,86,87]. It may facilitate the colonization of new environments by allowing species to adjust germination time in variable or new habitat schemes [88]. Thanks to laboratory research, much of which includes the analysis of mutants and changes in their dormancy and germination, the basic mechanisms responsible for the regulation of dormancy and germination are well known [6,22,89,90,91]. The mechanisms that regulate these processes are mainly based on maintaining a dynamic balance between abscisic acid (ABA) and gibberellins (GAs) and a set of many genes that regulate their metabolism, perception, and sensitivity through signaling networks [92,93,94,95,96,97]. Important for these processes are genes of the *DOG* (Delay Of Germinatio) family [98,99,100]. Seed dormancy-specific loci, including the *DOG* genes, were identified by analyzing the quantitative trait locus (QTL) using the natural variability of *Arabidopsis* [98,101,102,103]. One of the *DOG* family genes (*DOG1*) has been characterized in detail, and its genetic role in seed dormancy and the importance of gene expression in the sensing and adaptation of the environment have been well documented [34,98,104,105]. However, we do not know everything about this gene yet [100]. Transcription of *DOG1* is initially low in developing seeds; however, it increases with dormancy acquisition and disappears completely in the initial germination phase [105]. Consequently, the expression of *DOG1* shows a high correlation with the depth of seed dormancy [105]. For DOG1 protein, its chemical property, rather than quantity, is critical for maintaining seed dormancy, and its change into the nonfunctional form during maturation allows seed germination [100,106]. DOG1 protein, however, loses its function in completely nondormant seeds, which may be affected by the post-translational modification (PTM) of the protein [106]. The expression of *DOG1* is regulated by ABA [99], and both are necessary for determining primary seed dormancy [98,106,107]. However, *DOG1* may act independently of ABA to delay the germination of dormant seeds [108].

### 3.1. Physiological Control of Seed Dormancy and Germination

Despite the intense interest in adaptation to environmental shift, the primary characteristic of natural variation in germination is almost completely unknown. Especially, the physiological mechanisms of germination regulation vary in natural populations and how they are associated with responses to particular environmental factors are not known. Rodríguez et al. [22] pointed out that modulation by environmental cues affects the hormonal control of seed dormancy. Barua et al. [109] tested genetic variation in germination responses to distinct environmental factors, and examined the physiological mechanisms associated with those responses, including sensitivity to GA and ABA. They found that genetic variation for germination was environment-dependent. Hormonal sensitivities exhibited significant genetic variation. GA sensitivity was associated with germination responses to a variety of environmental factors, but ABA sensitivity was associated with particular germination responses, suggesting that an evolutionary change in GA sensitivity could affect germination in different environments, but that of ABA sensitivity may affect germination under more limited conditions. The physiological mechanisms of germination responses to particular environmental factors accordingly can influence the ability to adapt to different seasonal environments experienced during the colonization of new habitats or with future predicted climate change.

Seasonal behavior is relevant to fitness in temperate environments but it is unclear how offspring gain their initial seasonal characteristics. Plants use temperature signals to measure the time of year, and changes to life histories are therefore an important consequence of climate change. Chen et al. [110] showed that, in *Arabidopsis*, the current and past temperature experience of the mother plant is used to control the germination of progeny seeds via activation of the florigen *FLT* (Flowering Locus T) in fruit tissues. They demonstrated that maternal prior and current temperature experiences are transduced to the *FLT* locus in silique phloem. *FLT* controls seed dormancy by inhibiting proanthocyanidin biosynthesis in fruits, resulting in changed seed coat tannin content. These data show that maternal temperature history is integrated through *FLT* in the fruit to generate a metabolic signal that regulate the behavior of seeds according to the time of year. Chiang et al. [111] documented that *FLC* (Flowering Locus C) also regulates transition of seed germination, and the natural variation at the *FLC* locus and expression are associated with natural variation in temperature-dependent germination. *FLC*-mediated germination acts through *FT*, *SOC1* (Suppressor of Overexpression of Constans 1)*,* and *AP1* (Apetala1) genes in the flowering pathway before involving the ABA catabolic pathway (through Cytochrome P450 Monooxygenase—*CYP707A2*) and GA biosynthetic pathway (through Gibberellin 20-Oxidase 1—*GA20ox1*). Moreover, *FLC* regulation of germination is mostly maternally controlled, with *FLC* increasing and *FLT*, *SOC1*, and *AP1* levels declining at the late stages of seed maturation. Upregulation of *FLC* expression during seed maturation is associated with altered expression of *CYP707A2* and *GA20ox1* genes in germinating seeds, indicating that gene expression before the physiological independence of seeds can influence gene expression well after any physical connection between maternal plants and seeds exists. These observations suggest that *FLT* and *FLC* are part of the mediating mechanism in the plasticity of seed behavior. These genes mediate the environmental contribution during flowering and germination, suggesting the occurrence of the genetic mechanisms that control plant development in response to the environment [110,111].

### 3.2. Molecular Control of Seed Dormancy and Germination

The seasonal association of seed germination determines a plant’s realized environmental niche, and is important for climate adaptation [112]. The timing of seasonal germination depends on patterns of seed dormancy and release or induction by cold, and interacts with flowering time variation to construct different seasonal life histories. Analysis of the 559 accessions of the annual *Arabidopsis* from across a wide climate range showed natural variation in seed responses to chilling that correlated with flowering time and senescence [112]. Genome-wide association studies have identified several loci associated with natural variation in seed chilling responses, including a functional polymorphism in *DOG1*. A phylogenetic study of *DOG1* haplotypes indicated ancient divergence of these functional variants associated with periods of Pleistocene climate change, and revealed that allele turnover of candidate single-nucleotide polymorphisms (SNPs) was significantly associated with climate conditions. These results provide evidence that the germination niche and correlated life-history syndromes of *A. thaliana* are shaped by past climate changes, as is local adaptation to the contemporary climate [112]. Postma and Ågren [113] indicated that, with *DOG1*, the major QTL for seedling establishment is collocated and that selection has a significant role in the fitness advantage of local genotypes. This suggests that seed dormancy and *DOG1* are of high importance for explaining the variability associated with adaptation to local climate.

The above data show that depth of dormancy is determined genetically, but the environmental conditions experienced by the mother plant have a significant impact on the characteristics and efficiency of the seeds produced [10,19,26]. It was shown that different phytochromes contributed to germination, depending on seed maturation conditions [114]. Functional phytochromes PHYB and PHYD were necessary to break cool-induced dormancy, and PHYA contributed to the maintenance of dormancy. Effects of seed maturation temperature were much stronger than effects of seed maturation photoperiod [114]. Seasonal temperature changes are the main factor during seed ripening, affecting the depth of seed dormancy during the year [1,21,83,115,116] through the influence on the quantitative expression of *DOG1* [83,106,115]. Studies have been suggested that *DOG1* plays an important role in the adaptation of dormancy to the climate [104] and to local habitats [113]. The model of *A. thaliana* showed that the expression of the *DOG1* gene is increased and is associated with an increased degree of dormancy when the seeds mature at low temperatures [84]. Moreover, such a combination may yield a quantitative molecular link between maternal temperatures and future seed behavior [33,83,117]. It was also observed that *DOG1* overexpression increases seed germination sensitivity to inhibition by warm temperatures [108,118]. Thermoinhibition of seed germination is dependent on *DOG1* and does not involve an increased amount of ABA, which indicates that they work in parallel interacting pathways [119]. PHYD prevents the establishment of secondary dormancy caused by high temperature by efficient removal of the germination repressor PIL5, allowing GA accumulation and seed germination [120]. The analyses of He at al. [121] revealed that the effects of the parental temperature and nitrate environments were reflected by partly overlapping genetic and metabolic networks. Nitrogen metabolism-related metabolites (asparagine, γ-aminobutyric acid, and allantoin) were significantly decreased in both low temperature and low nitrate maturation environments. Nitrogen metabolism genes (Allantoinase, Nitrate Reductase 1, Nitrite Reductase 1, and Nitrilase 4) were differentially regulated in the low temperature and nitrate maturation environments. High light intensity during seed maturation increased galactinol content. Low light had an effect on cell surface-encoding genes in the near-*DOG6* isogenic line. Overall, the integration of phenotypes, metabolites, and transcripts led to new insights into the regulation of seed performance [121].

The identification of genes underlying dormancy QTLs is a major scientific challenge, which is relevant to ecological goals. Xiang et al. [122] described the identification of the *DOG18* QTL, which was identified as a factor in natural variation for seed dormancy in *Arabidopsis* [102]. *DOG18* encodes a 2C protein phosphatase involved in ABA-dependent dormancy, which was previously identified as the *RDO5* (Reduced Dormancy 5) gene [123]. *DOG18*/*RDO5* shows a relatively high frequency of loss-of-function alleles in natural accessions restricted to northwestern Europe. The release of dormancy in these alleles can be compensated by genetic factors such as *DOG1* and *DOG6*, and by environmental factors such as low temperatures experienced by the mother plant during seed maturation. RDO5 functions as a phosphatase that influences the seed phosphoproteome [122].

Depth of dormancy and gene expression patterns correlate with seasonal changes in soil temperature [33]. The germination potential of *Arabidopsis* seeds in soil shows that these seeds go through dormancy cycling and that the dynamics are genotype-dependent [124]. Dormancy cycling is driven by temperature, and the endosperm is important in the reception of the environment. RNA sequencing (RNA-seq) analysis has revealed that genes upregulated in the low- to nondormant stages are enriched for genes involved in translation, indicating that nondormant seeds are prepared for rapid seed germination. Some intriguing adaptive differences have been shown for the transcription profiles of *DOG1* in winter (Cvi) and summer (Bur) annual *A. thaliana* ecotypes, which were differentially correlated with the soil temperature cycle [27,33,34,105]. The dormancy level was correlated with the *DOG1* profile in Cvi, but not in Bur. This correlation suggests a plastic relationship between thermal sensing and the dormancy state. Plasticity results from the allelic variability in *DOG1*, and contributes to adaptation [83,104].

It has been indicated that when soil temperature is reduced in winter, dormancy increased as the expression of the ABA synthesis (via *NCED6*) and GA catabolism (via *GA2ox2*) genes increased [27]. This was linked to an increase in endogenous ABA. The expression of SNF1-related protein kinase genes, *SnrK2.1* and *SnrK2.4*, also increased, consistent with the modulation of enhanced ABA signaling and sensitivity by seasonal soil temperature. Increased temperatures in spring and summer caused declined dormancy; concurrent with this, decreased endogenous ABA along with increased gene expression of ABA signaling (ABA-Insensitive 2 (*ABI2*) and *ABI4*), ABA catabolism (*CYP707A2*), and GA synthesis (*GA3ox1*) were observed. During the low-dormancy phase in the summer, the expression of transcripts for the germination DELLA repressors *RGA* and *RGL2* was increased. Then, temporal separation of mechanisms occurs, with deep dormancy in winter controlled by ABA signaling, and shallow dormancy in spring and summer promoted by the repression of GA signaling [27]. Thus, seeds stay dormant throughout, but most importantly, the deep ABA-regulated dormancy is unresponsive to spatial signals such as light and GA, while the shallow dormancy due to DELLA repression is rapidly removed by exposure to light. This suggests that the switch to shallow dormancy enables a response to spatial signals such as light. This precise mechanism, well adapted to the conditions of a given climatic zone, can be easily exposed to perturbations in the conditions of climatic changes.

Footitt et al. [125] confirmed the hypothesis that components of the circadian clock may be involved in coordinating the annual seed dormancy cycle in soil seed banks. It was also found that the mechanism of response to changing environmental conditions also involves the following genes: Cbl-Interacting Protein Kinase 23 (*CIPK23*), Mother of flowering time (*MFT*) (in response to temperature), *NRT1.1* (in response to soil nitrate content), and *PHYA* (in response to light) [34]. *MFT* is strongly upregulated by low temperatures during seed maturation [126]. Loss of *MFT* leads to lower dormancy, indicating that *MFT* is a germination inhibitor, and variation of *MFT* underlies variation in seed dormancy [127]. The data show that *MFT* is a low-temperature-induced inhibitor of seed germination. The role of MFT as a germination inhibitor is conserved and *MFT* is a direct target of the SPATULA bHLH transcription factor [128,129]. *MFT* expression is strongly upregulated by the germination-inhibiting hormone 12-oxo-phytodienoic acid (OPDA), suggesting the involvement of oxylipin hormones in temperature responses in seeds [53,130]. MFT acts to control seed dormancy in a complex gene network including ABI5, DELLAs, and hormone metabolism [130].

### 3.3. Epigenetic Control of Seed Dormancy and Germination

Plants respond to environmental stimuli by activating signaling pathways that rapidly modify the rate of transcription of responsive genes and induce physiological responses [131,132]. For terrestrial plants that are inevitably exposed to daily and seasonal environmental changes, development reprogramming and response to environmental stimuli are particularly important [131,133]. Environmental conditions can induce adaptive epigenetic responses in plants and such effects can persist for generations [134]. Epigenetic processes play a role in the regulation of many key temperature-responsive genes in seeds, including *DOG1* and *FLC* [117,135]. Transcription regulation involves a multitude of regulatory layers, one of which is histone PTM. Histone modifications are implicated in influencing gene expression and genome function by establishing global chromatin environments and orchestrating DNA-based biological processes [136]. Chromatin organization is very strongly influenced by environmentally associated reprogramming processes [131,137]. The acetylation of specific lysine residues and methylations at lysine and arginine residues of histones play key roles in regulating gene expression. The promoter regions of active genes have reduced nucleosome occupancy and elevated histone acetylation and histone 3 lysine 4 (H3K4) methylation, whereas elevated levels of H3K27 methylation correlate with gene repression [136]. The monomethylations of H3K27, H3K9, H4K20, H3K79, and H2BK5 are all linked to gene activation, whereas trimethylations of H3K27, H3K9, and H3K79 are linked to repression [136]. Much research data document the importance of chromatin remodeling in the germination process. The trimethylation of histone H3K4 plays a role in ABA-driven seed germination inhibition through the regulation of ABA-Hypersensitive Germination 3 (*AHG3*) expression [138]. Wolny et al. [139] indicated that histone H3 and H4 acetylation patterns were more dynamic than those of DNA methylation in *Brachypodium distachyon* embryos during seed maturation and germination. Analysis of seed dormancy cycling in *Arabidopsis* according to chromatin remodeling showed changes in trimethylated H3K4 (H3K4me3) and H3K27me3 on *DOG1* in response to seasonal environmental signals [140].

Analysis of the expression of genes involved in chromatin remodeling via histone 2B (H2B) during dormancy cycling in the seed bank showed that expression of the gene-silencing repressor Repressor of Silencing1 (*ROS1*) correlated positively with dormancy, while the reverse was observed for the repressive histone methyl transferase Curly leaf (*CLF*) and the promoter of silencing Kryptonite (*KYP/SUVH4*) [140]. It was proposed that *ROS1*-dependent repression of silencing and a sequential requirement of *CLF*- and *KYP/SUVH4*-dependent gene repression and silencing are substantial for maintaining and suppressing dormancy. Changes in the histone marks H3K4me3 and H3K27me3 on *DOG1* were associated with seasonal environmental signals. During reduction in dormancy, H3K27me3 repressive marks slowly accumulated and accelerated upon exposure to light, completing dormancy loss. The marks on *DOG1* serve as a thermal sensing mechanism during dormancy cycling in preparation for light repression of dormancy. The authors concluded that the epigenetic mechanism, as with chromatin remodeling, plays a vital role in temporal sensing through the regulation of gene expression [140]. It was also indicated that a chromatin-remodeling factor, Pickle (*PKL*), represses the expression of embryonic traits during germination [141]. GA promotes the repression of seed-associated traits in *pkl Arabidopsis* seedlings [142]. *PKL*-dependent genes are enriched for trimethylation of H3K27. The histone deacetylases HDA6 and HDA19 regulate germination via the repression of embryo-specific genes, including *LEC1*, *FUS3*, and *ABI3* [143,144]. Other studies suggest the important role of the H2A.Z histone variant in the germination process [145]. In plants, the H2A.Z histone is located not only in the spool at the beginning of each gene, but are also especially frequent along the genes responding to environmental factors. Plants with a limited quantity of H2A.Z histone germinated much later than wild-type plants because they could not properly coordinate the activation and deactivation of genes in response to environmental factors [145]. All this suggests that changes in chromatin structure can be indicators of gene expression (de)activation under environmental signals.

*DOG1* acts by influencing the production of microRNAs (miRNAs) that govern the progression of developmental transitions through the plant life cycle, providing a molecular mechanism for the coordinated adaptation of seed dormancy to environmental conditions [119]. It has recently been shown that *DOG1* regulates seed dormancy by affecting miR-156 and miR-172 [119]. These miRNAs direct progression through the transition from dormancy to germination and indicate the potential mechanism of action of *DOG1*. In *Arabidopsis*, higher miR-156 levels led to increased seed dormancy [119]. Suppression of *DOG1* expression enabled seed germination in association with reduced miR-156 and increased miR-172 levels. The results reveal a link between the critical developmental phase transitions in the plant life cycle through a *DOG1*–miR-156–miR-172 interaction. In general, the data suggest that *DOG1* may transduce the environmental effect and indicate that successive changes in *DOG1* regulation at the chromatin level are closely related to environmental signals in the soil seed bank. This is consistent with the hypothesis that *DOG1* significantly affects the sensitivity of the process to environmental signals [118]. DOG1 protein undergoes PTMs during after-ripening, implying that DOG1 is regulated by PTMs related to reactive oxygen species (ROS) and nitric oxide (NO), such as *S*-nitrosylation and cysteine oxidation, and might be the hub regulator integrating environmental signals [6,106,146,147].

Recent data show that *DOG1* is widely regulated, with an antisense transcript (*asDOG1*) inhibiting its expression in seeds [148]. *asDOG1* is present in high levels in mature plants, where it inhibits *DOG1* expression under standard growth conditions. Inhibition was released by shutting down antisense transcription, which was triggered by ABA and drought. Loss of *asDOG1* resulted in high-level *DOG1* expression, conferring increased drought tolerance, while inactivation of *DOG1* caused enhanced drought sensitivity. The new role of *DOG1* in the environmental adaptation of mature plants is separate from its function in seed dormancy regulation. The demand of *asDOG1* to respond to ABA and drought demonstrates that antisense transcription is essential for sensing and responding to environmental changes in plants [149].

## 4. Conclusions

Due to climate change, the temporary regulation of germination is important and can be crucial for the plant to find an environmental window to prevent it from being exposed to stressful conditions that will lead to the death of nonadapted plants. The extraordinary event in the life history of seed plants was the evolution of seed dormancy, and various types and classes of dormancy reflect the adaptation of plants to various climatic and habitat conditions [1,84,148,150]. Seed dormancy broadens the success of seed germination in different environments [151]. Global warming contributes significantly to plant reproduction causing changes in ecosystems [75,152]. The adaptive variability of plasticity of the seed dormancy phenotype has a strong impact on the adaptation of plants to a changing climate [8]. An increasing number of papers present the results of research on a mechanism of adaptation of germination to environmental conditions at the level of gene expression. When combined, ecology and molecular biology offer the possibility of describing processes not at one, but at many different levels, building an overall model of organisms’ functionalities. Integrating data at several levels of regulation of the ecophysiological processes associated with seed dormancy breaking and germination could provide novel insight into this area of research concerning the life cycle of plants. This broad approach could yield valuable results for seed science, especially regarding further characterization of physiological and molecular components of environmentally regulated seed germination.
